# Assessment of tooth discoloration induced by root canal filling materials in pediatric dentistry

**DOI:** 10.1590/0103-6440202405838

**Published:** 2024-09-13

**Authors:** Fabiana Vitória Ananias Gonçalves, Orlando Aguirre Guedes, Sávio Akio Kachiyama, Larissa Pinzan Flauzino, Aurélio Rosa da Silva, Andreza Maria Fábio Aranha

**Affiliations:** 1 Department of Integrated Dental Science, School of Dentistry, University of Cuiabá, Cuiabá, Mato Grosso, Brazil.; 2 Department of Endodontics, School of Dentistry, Evangelical University of Goiás, Anápolis, Goiás, Brazil

**Keywords:** Primary tooth, tooth discoloration, root canal filling material, pediatric dentistry

## Abstract

This study investigated the potential for tooth discoloration of root canal filling pastes used in pediatric dentistry. Sixty bovine incisors were sectioned 2 mm apical to the cementoenamel junction and allocated into 6 groups (n = 10) according to the type of filling material used: G1- Zinc oxide-eugenol sealer; G2- Zinc oxide-eugenol and iodoform paste; G3- Calcium hydroxide (CH) and zinc oxide paste; G4- CH, zinc oxide, and iodoform paste; G5- CH and iodoform paste; and G6- Control. Polyethylene glycol 400 was used as a vehicle for CH-containing pastes. Color measurements were taken at specific intervals: preceding endodontic treatment (T0) and at successive points of 1 month (T1), 2 months (T2), 3 months (T3), and 1 year (T4) after the placement of the filling material. The color change (∆E) was calculated using the CIELab formula. Statistical analysis was performed using ANOVA, followed by Tukey's post hoc test (α = 5%). Significant differences were observed among the filling materials and time intervals (p <0.001). All groups exhibited color changes over time, except G1 and G5, which showed color changes only after 1 year. G1 and G2 demonstrated the highest ∆E values, with a statistically significant difference observed only at T2 when compared to G3 (p = 0.008). Root canal filling materials used in primary teeth have the potential to induce tooth discoloration.

## Resumo

Este estudo investigou o potencial de descoloração dentária de pastas de obturação de canal radicular utilizadas em Odontopediatria. Sessenta incisivos bovinos foram seccionados 2 mm apicais à junção amelocementária e distribuídos em 6 grupos (n = 10) de acordo com o tipo de material obturador utilizado: G1 - Pasta de óxido de zinco e eugenol; G2 - Pasta de óxido de zinco e eugenol e iodofórmio; G3 - Pasta de hidróxido de cálcio (HC) e óxido de zinco; G4 - de HC, óxido de zinco e iodofórmio; G5 - Pasta de HC e iodofórmio; e G6 - Controle. O polietileno glicol 400 foi utilizado como veículo nas pastas contendo HC. A mensuração da cor foi realizada em intervalos específicos: antes do tratamento endodôntico (T0) e nos pontos sucessivos de 1 mês (T1), 2 meses (T2), 3 meses (T3) e 1 ano (T4) após a inserção do material obturador. A alteração de cor (∆E) foi calculada utilizando a fórmula CIELab. A análise estatística foi realizada utilizando ANOVA, seguida pelo teste post hoc de Tukey (α = 5%). Diferenças significativas foram observadas entre os materiais de obturação e os intervalos de tempo (p < 0,001). Todos os grupos apresentaram alteração de cor ao longo do tempo, exceto G1 e G5, que mostraram mudanças de cor somente após 1 ano. G1 e G2 demostraram os maiores valores de ∆E, com diferença estatisticamente significativa observada apenas em T2 em comparação com G3 (p = 0,008). Materiais de obturação de canal radicular utilizados em dentes decíduos têm o potencial de induzir o manchamento dentário.

## Introduction

Tooth discoloration after endodontic treatment is an unpleasant complication ^1^ ^,^ ^2^ ^,^ ^3^ ^,^ ^4^, attributed to the composition of the material used ^4^, the cervical limit of the obturation (i.e., the presence of remnants of filling material in the pulp chamber) ^2^ ^,^ ^3^ ^,^ ^5^ ^,^ ^6^, and the time of interaction between the material and tooth tissue ^1^ ^,^ ^4^ ^,^ ^7^ ^,^ ^8^.

Several criteria should be considered during the selection of a material for endodontic treatment of primary teeth ^9^ ^,^ ^10^. Solubility, biocompatibility, radiopacity, antimicrobial potential, osteoinductive capacity, ability to provide a hermetic seal, and ease of manipulation and placement are some of the characteristics considered ideal for a filling material ^9^ ^,^ ^11^. In addition, great attention has been paid to the aesthetic factor ^8^ ^,^ ^11^, which is of great importance in dental treatment, particularly concerning the appearance of a smile ^12^.

Alteration in tooth color and consequently in smile harmony can negatively impact self-image, which may lead to emotional, social, and quality-of-life challenges in the pediatric population ^13^. The desire for improved smile aesthetics has become a decisive factor in seeking dental treatment ^13^.

The establishment of a consensus on the ideal filling material for primary teeth has been hampered by the limited amount of information available ^10^. Even so, four classes of filling materials have been commonly recommended: zinc oxide and eugenol paste (ZOE), iodoform pastes (IP), calcium hydroxide-based pastes (CH), and the combinations of these materials ^9^. Despite the numerous investigations on the discoloration of permanent teeth promoted by endodontic sealers ^1^ ^,^ ^2^ ^,^ ^5^ ^,^ ^11^, there is a noticeable scarcity of studies regarding tooth discoloration in primary teeth ^8^. Understanding the behavior of different filling materials used in endodontic therapy of primary teeth is decisive for an aesthetically satisfactory dental treatment. Therefore, this study aimed to assess the potential for tooth discoloration of root canal filling pastes commonly used in pediatric dentistry. The null hypothesis tested was that tooth discoloration does not vary based on (i) the type of filling paste and (ii) the duration of contact with the tooth structure.

## Material and methods

### Specimen preparation

Sixty bovine incisors with standardized features, complete root development, and crowns without enamel defects were selected. All teeth were cleaned, and their roots were sectioned 2 mm below the cementoenamel junction (CEJ) using a precision saw (Isomet 1000; Buehler Ltd, Lake Bluff, IL, USA). The apical access was widened using #2135F bur (KG Sorensen, Barueri, SP, Brazil). Next, the pulp chambers were emptied using K #40 files (Maillefer Dentsply Sirona, York, PA, USA) and abundant irrigation with 0.9% saline solution. The specimens were stored in distilled water at 37°C ^3^ ^,^ ^14^.

### Root filling pastes

The specimens were randomly divided into 1 control group (no filling; n = 10) and 5 experimental groups (n = 10) via a random number sequence generator. In Box 1, the filling materials, their respective manufacturers, and handling formulas ^15^ ^,^ ^16^ are presented. For each evaluated filling material, an attempt was made to achieve a consistency similar to that of a dentifrice, recommended to facilitate the insertion and sealing of the root canal ^8^ ^,^ ^15^.

**Box 1:**
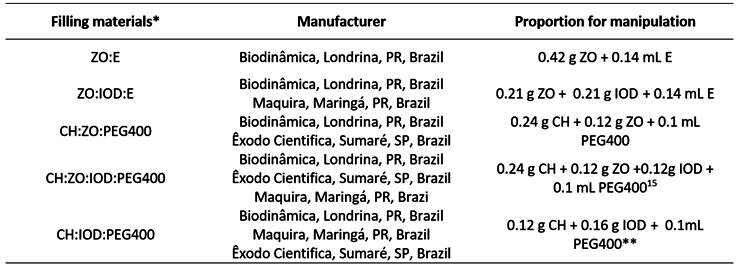
Distribution of experimental groups according to root filling pastes: composition, manufacturer, and handling proportion.

*ZO:E - Zinc oxide eugenol sealer; ZO:IOD:E - Paste of zinc oxide, iodoform and eugenol; CH:ZO:PEG400 - Paste of calcium hydroxide, zinc oxide and poly (ethylene glycol) 400; CH:ZO:IOD:PEG400 - Paste of calcium hydroxide, zinc oxide, iodoform and poly (ethylene glycol) 400; CH: IOD:PEG400 - Paste of calcium hydroxide, iodoform and poly (ethylene glycol) 400.

** The concentrations of IOD and CH used were the same as those present in the commercial paste Vitapex (Neo Dental International, Inc, Federal Way, WA, USA).

### Pulp chamber filling

The pulp chambers were dried, and the tested filling materials were inserted until the cementoenamel junction (CEJ) using a 3 mL plastic syringe (BD SoloMed, Curitiba, PR, Brazil), Precision #4 tips (Maquira, Maringá, PR, Brazil), and Lentulo drill #4 (Maillefer Dentsply Sirona). Excess material was removed, and the cavities were sealed with Filtek Z350 composite resin (3M ESPE do Brasil Ltda, Sumaré, SP, Brazil), following a two-step adhesive protocol - Single Bond 2 (3M ESPE).

### Spectrophotometric analysis

The spectrophotometric analysis was conducted at the following evaluation period: T0 - after emptying the pulp chambers (baseline); T1 - 1 month after obturation; T2 - 2 months after obturation; T3 - 3 months after obturation; and T4 - 1 year after obturation.

Tooth color assessment was performed using a VITA Easyshade® spectrophotometer (VITA Zahnfabrik, Bad Säckingen, Germany), previously calibrated according to the manufacturer's instructions. using the inbuilt calibration block (white color). The spectrophotometer was recalibrated after measuring five specimens. All measurements were performed by the same operator. The equipment provides color data based on three dimensions, as recommended by the International Commission on Illumination ^17^, represented by the parameters L*, a*, and b*. The L* parameter (luminosity) ranges from 0 to 100, encompassing from absolute black to absolute white. On the other hand, the a* and b* parameters represent chromatic directions ranging from red (+a) to green (-a) and from yellow (+b) to blue (-b), respectively.

For each specimen, the color variation (ΔE) was calculated about the initial color (T0), using the CIELab formula,2 ^3^:

$${{∆E}^{*}}_{ab}={\left[{\left({∆L}^{*}\right)}^{2}+{\left({∆a}^{*}\right)}^{2}+{\left({∆b}^{*}\right)}^{2}\right]}^{1/2}$$

$${∆L}^{*}=\left(L_{1}^{*}-L_{o}^{*}\right);{∆a}^{*}=\left(a_{1}^{*}-a_{0}^{*}\right);{∆b}^{*}=\left(b_{1}^{*}-b_{0}^{*}\right)$$

The parameter ΔEab represents the color variation, where ΔL is the difference in luminosity (L_1_ - L_0_), Δa is the difference along the a axis (a_1_ - a_0_), and Δb is the difference along the b axis (b_1_ - b_0_). The data L_0_, a_0_, and b_0_ correspond to the initial readings (baseline), while L_1_, a_1_, and b_1_ represent the data from the readings at each evaluation period.

For evaluation, the specimens were positioned in a standardized mode. The crowns were individually fixed onto wax support (7 cm x 3 cm x 2 cm) (Lyzanda, São Paulo, SP, Brazil) within a prefabricated wooden box (4 cm x 4 cm x 2.5 cm) with equidistant holes to accommodate the tip of the spectrophotometer ^3^ ^,^ ^11^ ^,^ ^14^. During the evaluation, readings were taken in three equidistant areas of the crown of each specimen. For each area, two readings were taken, totaling six readings per specimen. The mean ΔE was then calculated for each specimen at each evaluation period. During the spectrophotometric analysis, the crowns remained moist to prevent any chromatic alterations due to drying ^18^.

### Statistical analysis

To determine the sample power, four premises were considered: a relevant difference in color variation (∆E > 3.7) ^14^, a 5% α error, a standard deviation of 2, and a test power of 80%. A one-way analysis of variance (ANOVA) was performed (p < 0.05).

The mean ΔE values in the different groups and investigated evaluation periods were subjected to repeated measures ANOVA and Tukey's post hoc test, with a significance level of 5%, using the statistical software Minitab® 19 (Minitab, State College, PA, USA).

## Results

The statistical analysis indicated a significant influence of both the type of root-filling paste (p <0.001) and the evaluation period (p <0.001) on tooth discoloration. However, when examining the relationship between the filling material and evaluation period (p = 0.981), no significant effect was observed (Table 1).

Table 2 presents the L*, a*, and b* values for the tested filling pastes at various time intervals. All groups demonstrated color comparability as no statistically significant difference was observed in the three parameters at the baseline. L* and a* values differed between the groups only at the 1-year observation time, while the b* values varied after 2 and 3 months. Assessing each group individually, it was found that the a* values changed significantly over time in G1 and G2, whereas the b* values demonstrated this trend in all tested groups except for G5.

The ΔE values are presented in Table 3. All groups exhibited a change in tooth color over time, except G1 and G5, both demonstrated increased darkening only after 1 year. When comparing the groups within each evaluation period, a significant difference between G1 and G2 compared to G3 was observed, indicating a more pronounced tooth color change.

**Table 1:** Effect of time interval and filling material and their association on tooth discoloration.

Variation	Sum of squares	P value*
Root canal filling material	432.7	< 0.001
Time interval	700.0	< 0.001
Root canal filling paste _*_ Time interval	62.3	0.981

*Repeated measure ANOVA; P<0.05

**Table 2:** L*, a*, b* values (CIE) for the tested filling pastes at different time intervals.

Filling materials*		Time intervals (∆E Mean ± SD)**			
	Baseline	1 month	2 months	3 months	1 year
	L_0_	L_1_	L_2_	L_3_	L_4_
ZO:E	L_0_	88.8 ± 3.9^A,a^	87.6 ± 4.6^A,a^	85.6 ± 6.2^A,a^	81.3 ± 9.0^B,a^
ZO:IOD:E	89.4 ± 3.9^A,a^	89.8 ± 2.8^A,a^	90.1 ± 3.3^A,a^	88.9 ± 3.9^A,a^	90.6 ± 3.1^A,a^
CH:ZO:PEG400	89.8 ± 4.6^A,a^	89.4 ± 1.8^A,a^	89.8 ± 3.8^A,a^	91.0 ± 1.9^A,a^	89.4 ± 4.2^AB,a^
CH:ZO:IOD:PEG400	89.8 ± 4.0^A,a^	88.6 ± 2.7^A,a^	89.1 ± 2.4^A,a^	90.6 ± 3.1^A,a^	91.7 ± 2.8^A,a^
CH:IOD:PEG400	89.4 ± 3.9^A,a^	88.3 ± 5.1^A,a^	89.1 ± 4.9^A,a^	89.1 ± 5.9^A,a^	90.2 ± 6.1^A,a^
Control	87.8 ± 4.5^A,a^	89.2 ± 1.7^A,a^	89.4 ± 3.6^A,a^	88.0 ± 1.9^A,a^	88.7 ± 4.2^AB,a^
	89.1 ± 3.9^A,a^	a_1_	a_2_	a_3_	a_4_
ZO:E	a_0_	0.9 ± 2.2^A,a^	2.4 ± 1.6^A,ab^	2.7 ± 2.0^A,ab^	4.5 ± 2.8^A,b^
ZO:IOD:E	0.9 ± 2.9^A,a^	-0.3 ± 1.0^A,a^	0.7 ± 1.4^A,ab^	1.0 ± 1.6^A,ab^	1.8 ±1.3^B,b^
CH:ZO:PEG400	-0.3 ± 1.3^A,a^	0.8 ± 1.8^A,a^	1.7 ± 1.5^A,a^	1.1 ± 1.9^A,a^	2.2 ± 1.1^AB,a^
CH:ZO:IOD:PEG400	0.7 ± 2.3^A,a^	1.1 ± 1.8^A,a^	2.3 ± 1.5^A,a^	1.9 ± 1.7^A,a^	2.2 ± 1.1^AB,a^
CH:IOD:PEG400	1.3 ± 2.1^A,a^	-0.5 ± 0.8^A,a^	1.4 ± 2.0^A,a^	1.3 ± 2.2^A,a^	1.7 ± 2.1^B,a^
Control	-0.5 ± 1.1^A,a^	0.8 ± 1.8^A,a^	1.7 ± 1.5^A,a^	1.1 ± 1.9^A,a^	2.2 ± 1.1^AB,a^
	0.9 ± 2.4^A,a^	b_1_	b_2_	b_3_	b_4_
ZO:E	b_0_	15.9 ± 4.3^A,a^	21.9 ± 3.0^A,b^	22.0 ± 3.3^A,b^	24.8 ± 3.5^A,b^
ZO:IOD:E	15.6 ± 5.7^A,a^	13.2 ± 4.3^A,a^	17.8 ± 3.6^AB,ab^	18.3 ± 4.4^AB,ab^	25.1 ± 3.4^A,c^
CH:ZO:PEG400	13.0 ± 6.6^A,a^	16.0 ± 4.5^A,a^	17.4 ± 4.0^AB,ab^	17.4 ± 5.5^AB,ab^	23.2 ± 5.6^A,b^
CH:ZO:IOD:PEG400	15.7 ± 5.3^A,a^	14.7 ± 3.2^A,a^	18.4 ± 4.1^AB,ab^	17.3 ± 4.4^AB,ab^	22.5 ± 4.9^A,b^
CH:IOD:PEG400	14.3 ± 5.6^A,a^	14.3 ± 6.2^A,a^	16.4 ± 4.3^B,a^	15.4 ± 4.8^B,a^	20.4 ± 4.9^A,a^
Control		15.7 ± 4.5^A,a^	16.8 ± 4.0^AB,ab^	17.1 ± 5.5^AB,ab^	22.2 ± 5.6^A,b^

*ZO:E - Zinc oxide eugenol sealer; ZO:IOD:E - Paste of zinc oxide, iodoform and eugenol; CH:ZO:PEG400 - Paste of calcium hydroxide, zinc oxide and poly (ethylene glycol) 400; CH:ZO:IOD:PEG400 - Paste of calcium hydroxide, zinc oxide, iodoform and poly (ethylene glycol) 400; CH: IOD:PEG400 - Paste of calcium hydroxide, iodoform and poly (ethylene glycol) 400; Control (no filling). **Means with the same letter are not significant at p>0.05. Uppercase letters for comparisons across columns (intergroup comparisons); Lowercase letters for comparisons within rows (intragroup comparisons) (ANOVA with post- hoc Tukey; α=5).

**Table 3:** Mean and standard deviation (SD) for color change (∆E) for tested filling pastes at different time intervals.

Filling materials*	Time intervals (∆E Mean ± SD)				
	1 month	2 months	3 months	1 year	*P value*
ZO:E	7.97 ± 3.95^A,a^	8.44 ± 4.99^A,a^	9.13 ± 3.85^A,a^	13.32 ± 6.06^A,a^	0.067
ZO:IOD:E	5.99 ± 3.02^A,a^	7.42 ± 3.53^A,a^	8.56 ± 2.76^A,a^	12.55 ± 2.97^A,b^	<0.001
CH:ZO:PEG400	4.04 ± 1.29^A,ab^	3.13 ± 1.62^B,a^	5.92 ± 2.48^A,bc^	8.20 ± 2.44^A,c^	<0.001
CH:ZO:IOD:PEG400	5.30 ± 2.66^A,a^	5.71 ± 2.10^AB,a^	8.22 ± 3.35^A,ab^	9.58 ± 4.07^A,b^	0.011
CH:IOD:PEG400	6.3 ± 5.47^A,a^	6.89 ± 5.70^AB,a^	7.70 ± 5.32^A,a^	10.80 ± 5.52^A,b^	0.181
Control	7.83 ± 2.32^A,a^	10.93 ± 2.46^A,a^	7.43 ± 5.43^A,a^	7.54 ± 3.40^A,a^	0.060
*P value*	0.223	0.008	0.364	0.067	

*ZO:E - Zinc oxide eugenol sealer; ZO:IOD:E - Paste of zinc oxide, iodoform, and eugenol; CH:ZO:PEG400 - Paste of calcium hydroxide, zinc oxide and poly (ethylene glycol) 400; CH:ZO:IOD:PEG400 - Paste of calcium hydroxide, zinc oxide, iodoform and poly (ethylene glycol) 400; CH: IOD:PEG400 - Paste of calcium hydroxide, iodoform and poly (ethylene glycol) 400; Control (no filling). **Means with the same letter are not significant at p>0.05. Uppercase letters for comparisons across columns (intergroup comparisons); Lowercase letters for comparisons within rows (intragroup comparisons) (ANOVA with post- hoc Tukey; α=5%).

## Discussion

The current study assessed the potential for tooth discoloration of root canal filling materials used in pediatric dentistry. The results indicated that both the type of filling material and the evaluation period had a significant impact on tooth color change. Thus, the tested null hypothesis was rejected.

This study opted for a bovine teeth model due to the challenges in accessing human teeth for dental research. Bovine teeth offer advantages in handling owing to their size, enabling standardization of age and internal space, and mitigating the risk of disease transmission ^19^. Moreover, the use of bovine teeth allowed the consolidation of various features, reducing potential confounding factors associated with dentin. Despite minor micro and macrostructural differences between human and bovine teeth ^20^, previous studies consider bovine teeth a suitable model for tooth discoloration research ^8^ ^,^ ^11^.

Tooth color variation (∆E) was assessed using spectrophotometry due to its reliability, precision, and reproducibility ^1^ ^,^ ^2^ ^,^ ^3^ ^,^ ^5^ ^,^ ^8^ ^,^ ^11^ ^,^ ^14^ ^,^ ^18^. This method minimizes the impact of subjective variables, such as lighting conditions, examiner experience, age, gender, and visual fatigue. Consequently, it ensures a more objective and consistent evaluation of the color of the studied tooth tissue ^17^ ^,^ ^21^

One possible limitation of the current study was the use of the CIELab formula (∆Eab) for tooth color evaluation. This formula relies solely on the parameters of luminosity (L) and chroma (a* and b*), as specified by the CIE in 1978. In contrast, the CIEDE 2000 system, which incorporates parametric weighting of hue and integrates the visual assessment of the object (∆E00) ^5^ ^,^ ^8^ ^,^ ^22^, provides a more comprehensive approach ^17^, making it particularly well-suited for clinical applications ^17^. Including this system in future research would offer a more holistic understanding of color variation, enhancing the study’s applicability and relevance, especially in clinical contexts.

The analysis of tooth color variation (∆E) combines the thresholds of perceptibility and acceptability ^22^. The perceptibility threshold (LP) represents the color difference perceived by the human eye, with the CIELab system ranging from ∆Eab = 1.1 to 3.7 ^3^ ^,^ ^12^ ^,^ ^18^ ^,^ ^21^ ^,^ ^22^. On the other hand, the acceptability threshold (LA) represents the color difference considered clinically acceptable, which in Dentistry holds greater significance than the perceptibility threshold, ranging from ∆Eab = 2.7 to 6.8 ^12^ ^,^ ^21^ ^,^ ^22^.

All the root-filling pastes investigated in the present study exhibited ∆E_ab_ values exceeding 3.7, except G3 at T2, which is consistent with findings from previous studies ^3^ ^,^ ^12^ ^,^ ^18^. This indicates a clinically perceptible tooth color alteration, surpassing the ∆E_ab_ value of 1.8, as suggested by Paravina et al. ^22^. It's important to note that this study didn't specifically assess the clinical aspect of the crown.

Thirty days after the placement of filling materials, a change in tooth coloration (∆E) was observed. This finding is consistent with the results reported in previous studies ^1^ ^,^ ^2^ ^,^ ^3^ ^,^ ^14^ ^,^ ^18^. It's important to mention that the observed tooth color change occurred despite existing evidence suggesting that the presence of a smear layer might hinder the diffusion of chromogenic components of filling materials through dentinal tubules ^6^ ^,^ ^7^ ^,^ ^14^.

The ∆E values obtained in this study were notably higher compared to those reported by Xavier et al. ^8^, who evaluated the effects of filling materials for pulpotomies and pulpectomies in primary teeth. Factors such as the type of filling material, methodological design, and smear layer management may have contributed to these divergent outcomes.

Although not conclusive, the presence of eugenol in the composition of root canal filling pastes seems to contribute to the darkening of the tooth structure. In Group 3, the combination of calcium hydroxide, zinc oxide, and the PEG400 showed the lowest ∆E values across all evaluated periods. However, these values were not statistically different from the other pastes, except for G1 and G2 after 3 months. It's worth highlighting that both G1 and G2 contained eugenol in their composition.

G1 demonstrated noticeable changes in the L*, a*, and b* parameters, indicating a decrease in luminosity (L*) and a significant increase in chromatic values (a* and b*). These findings are in accordance with those of previous studies that observed a similar effect induced by zinc oxide eugenol-based endodontic sealer ^1^ ^,^ ^3^ ^,^ ^14^. From a clinical perspective, these findings suggested the likelihood of crown darkening and a discernible shift in color towards shades of orange-red and yellow ^1^ ^,^ ^3^ ^,^ ^6^ ^,^ ^14^. This phenomenon could be attributed to the chemical reaction of eugenol with fluids present in the dentin tissue, especially in a moist environment after setting, leading to the continuous release of eugenol and subsequent deposition of zinc hydroxide ^23^. This process, in turn, results in the gradual darkening of the dental structure over time ^1^. Further research and a deeper understanding of these chemical processes are warranted to validate this mechanism.

Although tooth discoloration caused by calcium hydroxide (CH) is minimal or even absent ^11^, a change in tooth structure coloration becomes evident when CH is associated with other components, such as iodoform ^8^ and zinc oxide ^2^ ^,^ ^6^ ^,^ ^7^ ^,^ ^8^ ^,^ ^11^. This phenomenon was also observed in the current study.

In a study conducted by Xavier et al. ^8^, an increase in the ∆E values of teeth filled with Vitapex paste, composed of CH and IOD, was observed after a 6-month evaluation period. This observation aligns with the findings of the current investigation for G2, G4, and G5, where iodoform was present in the filling pastes. It is plausible that the tooth discoloration occurred due to the decomposition of iodoform, a yellow crystalline solid ^24^. This decomposition can release small particles of iodine and other byproducts that, upon contact with dentin, have the potential to penetrate dentinal tubules, causing staining in the dentin structure ^25^.

It is noteworthy that the lowest ∆E values were consistently observed in G3 (CH and ZO) across all assessed periods. This contrasts with the findings in G1 and G2 after 2 months, which contradicts a previous study ^8^, where Calen paste thickened with ZO showed higher ∆E values at the 3-month mark. However, Xavier et al. ^8^ reported a decrease in ∆E at 6 and 9 months of evaluation, while our current study observed a significant increase in ∆E starting from the 3-month mark.

Also, in G3, the parameter L* exhibited statistically lower values compared to the other groups, resulting in a tendency towards a lighter gray appearance, consistent with previous reports ^6^. This occurrence can potentially be attributed to the presence of zinc oxide, which undergoes chemical interactions with oxygen molecules ^26^. These interactions occur within the dentin composition and lead to modifications in tooth coloration, although to a lesser degree when compared to substances like eugenol, bismuth oxide, silver powder, barium sulfate, and iodoform, which are prevalent in endodontic sealer ^2^ ^,^ ^6^ ^,^ ^7^ ^,^ ^8^ ^,^ ^11^.

It’s important to recognize that the present methodological model involved the filling of the pulp chambers instead of the root canals. This decision was based on the scarcity of research evaluating materials for pulpectomy of primary teeth, with only one study conducted by Xavier et al. ^8^. It is important to highlight that this study used a similar methodology, which may facilitate the result comparisons. However, it is essential to highlight that a more clinically relevant approach would involve evaluating the crown color changes induced by these materials during root canal filling. Thus, additional studies are needed to provide a comprehensive understanding of the color changes induced by different filling materials in pediatric dentistry. These studies would contribute significantly to guiding clinical practices in pediatric endodontics. In conclusion, root filling pastes used in pediatric dentistry induce dental color change.
